# Quality of life, sense of coherence and occupational balance one year after an occupational therapy intervention for people with depression and anxiety disorders

**DOI:** 10.3233/WOR-220096

**Published:** 2023-10-19

**Authors:** Petra Wagman, A. Birgitta Gunnarsson, Fredrik Hjärthag, Katarina Hedin, Carita Håkansson

**Affiliations:** a School of Health and Welfare, Jönköping University, Jönköping, Sweden; b Department of Research and Development, Region Kronoberg, Växjö, Sweden; c Department of Clinical Neuroscience and Rehabilitation, Gothenburg University, Gothenburg, Sweden; d Faculty of Arts and Social Sciences, Karlstad University, Karlstad, Sweden; e Futurum, Region Jönköping County, Jönköping, Sweden; f Department of Medical and Health Sciences, Linköping University, Linköping, Sweden; g Department of Clinical Sciences in Malmö, Family Medicine, Lund University, Lund, Sweden; h Division of Occupational and Environmental Medicine, Lund University, Lund, Sweden

**Keywords:** Mental health, rehabilitation, return to work

## Abstract

**BACKGROUND:**

Quality of life (QOL), sense of coherence (SOC) and occupational balance (OB) have been found to increase after rehabilitation among people living with depression and anxiety. However, these aspects have not been investigated over time in participants with different paid work situations, such as being on sick leave or not.

**OBJECTIVE:**

To describe and compare the self-rated QOL, SOC and OB after participation in occupational therapy in three groups of people with depression and anxiety disorders based on their work situation during the study period: continuous sick leave, return to work and continuous work.

**METHODS:**

Forty-seven women and seven men, 19–60 years old with depression and anxiety were followed over time. They completed questionnaires on QOL, SOC and OB on four occasions. Data were analyzed with non-parametric statistics.

**RESULTS:**

No statistically significant differences between the groups were identified. All groups improved in at least one of the studied areas over time: the sick leave group in OB; the return-to-work group in QOL and OB; and the Work group in QOL and SOC.

**CONCLUSION:**

The results indicate variations in QOL, SOC and OB between people with different work situations over time after occupational therapy. Studies with larger samples are, however, warranted.

## 1 Introduction

Mental health issues such as depression and anxiety disorders are common and have increased globally [1] and have significantly contributed to long-term disability in European countries [2] and are common causes for sick leave in Sweden [3]. People living with these disorders face many challenges in their everyday life [4–6]. Where paid work is concerned, participants in research studies have described the importance of work [7] but also problems related to maintaining their ability to work [7, 8]. Moreover, the reason for being on sick leave [9] as well as for returning to work are impacted by factors related to the individual and to his/her context at work and at home [10–12]. Improving the total life situation for people with depression and anxiety, including work, thus becomes important.

This group have been found to benefit from treatment such as occupational therapy [13–16], including a return to paid work [14, 16–18]. The present study is part of a larger project in which participants with depression and anxiety participated in occupational therapy and have been followed from prior to the start of this intervention to one year after its completion [19]. The focus in a previous study [20] from the project was on the participants’ perceptions of their performance of everyday activities and their satisfaction with their performance, as measured with the Canadian Occupational Performance Measurement [21], in relation to their paid work situation. The participants were divided into three sub-groups in the statistical analyses based on their paid work situation: on sick leave throughout the study period; on sick leave at baseline but returned to work to some extent during the year; and working throughout the study period. The result showed that all groups had difficulties with their performance of everyday activities as well as with their satisfaction with the performance at baseline. Furthermore, the participants in all groups improved their performance and/or satisfaction with their performance in some activity areas, but the number differed between the groups. The group on sick leave increased in two activity areas (socialisation and work), while the return-to-work group improved in three areas (active recreation, socialisation and work) and the working group improved in four areas (active recreation, household, personal care and socialisation) [20]. Further investigation of other health-related aspects of potential importance for work ability might generate valuable information in relation to the sample differing in their paid work situation. Quality of Life (QOL) has been found to increase in people with depression and anxiety disorders after occupational therapy treatment [13, 22–24]. However, this does not yet seem to have been studied separately in people who vary in terms of their paid work situation. Similarly, Sense of Coherence (SOC) [25] has been found to increase in people with depression and anxiety disorders after treatment [22, 26, 27]. The pre-rehabilitation level of SOC has also been found to predict the level of post-rehabilitation symptoms in this group [26], but no subgroup division based on variations of the paid work situation was conducted in these studies [22, 26, 27]. Higher levels of SOC have, however, been reported in other populations returning to work [28, 29]. This has also been reported in a study including a minority of participants having mental health issues [30], making it relevant to undertake further investigation in this population. Moreover, Occupational Balance (OB) i.e. the person’s perception of his/her total mix of activities in everyday life [31] is another aspect of potential relevance. OB have been found to increase in people with mental health conditions, including people with depression and anxiety disorders, after occupational therapy treatment [13, 14, 22, 23]. No study has been identified investigating OB among people with depression and anxiety varying in their paid work situation.

The present study focuses on following QOL, SOC and OB over time in people with depression and anxiety who participated in occupational therapy. The aim was thus to describe and compare the self-rated Quality of Life, Sense of Coherence and occupational balance in three groups of people with depression and anxiety disorders, based on their work situation during the study period: continuous sick leave, returned to work and continuous work.

## 2 Method

The study has a longitudinal design. The data came from a larger project focusing on the Tree Theme Method^®^ (TTM) as an intervention [19]. It took place in the context of primary healthcare and mental health outpatient clinics in southern Sweden (Clinical Trials.gov: NCT01980381). The project followed the principles of the Helsinki Declaration [32] and was approved by the Regional Ethical Review Board in Linköping, Sweden (2012/232-31 and 2015/12-32).

### 2.1 Participants

The inclusion criteria for the project were: being diagnosed with depression and/or anxiety disorders; being of a working age (18–65 years); and considered by their occupational therapist to have problems with their everyday activities. The exclusion criteria were: ongoing psychosis, severe somatic disorder, and/or severe difficulties participating in interviews or completing questionnaires. Additional inclusion criteria in the present study were: participation in all four data collections and either being on sick leave the whole period (the SL group), on sick leave at baseline but returning to work to some degree during the period (RTW group) or continuing working the whole period (the Work group).

### 2.2 Data collection and instruments

Each eligible participant was informed about the project. Those interested received further information at a meeting with a project assistant who informed of the voluntary nature of their participation, of the possibility of withdrawing and about confidentiality. They were also able to ask questions prior to signing their informed consent. They then answered questions of a socio-demographic nature including gender, age, education, having young children, and friends. The project assistants who collected the data were trained and validated occupational therapists (not involved in the participants’ treatment). The data collection occurred on four occasions: prior to occupational therapy treatment (T0), after completion of treatment (T1), 3 months after treatment (T2), and 12 months after treatment (T3). The baseline (T0) data collection started in 2013 (*n* = 118). The 12 months follow up data collection (T3) ended in 2017 (*n* = 84). Fifty-four of those participants met the additional criteria and were included in the present study.

The participants completed several instruments related to various aspects of health and everyday life during the four data collections. T0 data from the Hospital Anxiety and Depression Scale (HADS) [33, 34] was used for providing information about self-rated symptoms of depression and anxiety at baseline in the present study. Each of its subscales ranges 0–21, where higher ratings mean a higher level of severity of these conditions. Data on QOL, SOC and OB from all four data collections were also used.

QOL was measured with the *Manchester Short Assessment of Quality of Life (MANSA)* [35]. It covers different aspects of life, e.g., leisure and relationships, and includes a general question about satisfaction with life as a whole. The MANSA [35] comprises 12 items measured on a 7-point ordinal scale where higher scores indicate a better QOL and the summed score was used, ranging from 12–84. The Swedish version has shown good internal consistency and construct validity [36]. The Cronbach’s alpha in the present sample was 0.78 at baseline.

SOC was measured with the *Sense of Coherence* scale *(SOC)* [37] that comprises 13 items measured on a 7-point ordinal scale. The summed score was used, which ranges from 7-91, with a higher score indicating a better SOC. The Swedish SOC has demonstrated good test-retest reliability and internal consistency [38]. The Cronbach’s alpha in the present sample was 0.84 at baseline.

OB was measured with the *Occupational Balance Questionnaire (OBQ)* [39] which comprises 13 items measured on a 6-point ordinal scale. The summed score was used, which ranges from 0–65, where the higher score denotes a better occupational balance. The OBQ has shown good internal consistency [39–41] and sufficient test-retest reliability [39]. The Cronbach’s alpha in the present sample was 0.86 at baseline.

### 2.3 Data analysis

Descriptive statistics were used for analyzing the participants’ demographics and ratings of the included instruments. Potential differences between the groups were investigated with the Kruskal Wallis Test while the Friedman Test was used between different time points within each group. Multiple analyses were corrected for with Bonferroni correction. *P*-values<0.05 were considered significant. IBM SPSS Statistics version 25 was used.

## 3 Results

Forty-seven women and seven men between 19 and 60 years met the inclusion criteria. The SL group consisted of 24 participants, the RTW group 13 participants and, the Work group 17 participants (Table 1). One of the participants in the RTW had returned to work at T1, seven more at T2 and five at T3.

**Table 1:** Demographic characteristics of the participants with depression and anxiety disorder (*n* = 54) at baseline (T0)

	Sick leave (*n* 24)	Return-to-work (*n* 13)	Working (*n* 17)
Gender
Women *n* (%)	22 (92)	11 (85)	14 (82)
Men *n* (%)	2 (8)	2 (15)	3 (18)
Age (years)
Median (min–max)	51 (21–60)	42 (19–60)	39 (20–57)
Education
Mandatory school *n* (%)	8 (33)	2 (15)	1 (6)
High school *n* (%)	10 (42)	8 (62)	10 (59)
University *n* (%)	6 (25)	3 (23)	6 (35)
Having children under 18
Yes *n* (%)	6 (25)	3 (23)	3 (18)
Having friends
Yes *n* (%)	21 (88)	12 (92)	15 (88)
Symptoms (HAD*)
Depression (median)	12	10	7
Anxiety (median)	16	14	11

*HAD=Hospital Anxiety and Depression scale.

The groups did not differ significantly in QOL on any occasion. The RTW and Work groups increased significantly over time, Table 2.

**Table 2:** Quality of life (QOL) measured with the Manchester Short Assessment of Quality of Life from T0 to T3 in participants with depression and anxiety disorder

	QOL T0 Median Min–Max	QOL T1 Median Min–Max	QOL T2 Median Min–Max	QOL T3 Median Min–Max	*P*-value within samples^2^
Sick leave (*n* 24)	44 15–68	50^v^ 21–71	48 19–71	46^v^ 32–78	0.129
Return-to-work (*n* 13)	50 35–64	52 34–69	60 28–71	67 38–77	**0.003**
Working (*n* 17)	49 23–74	60 30–72	61 23–82	68 22–77	**0.027**
*P*-value between samples^1^	1.000	0.532	0.204	0.100

^1^Kruskal Wallis ^2^Friedman ^v^*n* = 23 **Bold** = Significant changes. T0 baseline, T1 after treatment, T2 3 months after treatment, T3 12 months after treatment. Bonferroni corrections have been conducted in relation to each analysis.

SOC did not differ significantly between the groups on any occasion. The Work group significantly increased their scores over time, Table 3.

**Table 3:** Sense of Coherence (SOC) measured with the Sense of Coherence Scale from T0 to T3 in participants with depression and anxiety disorder

	SOC T0 Median	SOC T1 Median	SOC T2 Median	SOC T3 Median	*P*-value within
	Min–Max	Min–Max	Min–Max	Min–Max	samples^2^
Sick leave (*n* 24)	45 19–72	47 26–73	47 22–80	46 34–79	1.000
Return-to-work (*n* 13)	46 31–62	52 31–80	60 29–84	66 31–87	0.513
Working (*n* 17)	55 32–79	59 38–85	60 34–83	60 37–86	**0.027**
*P*-value between samples^1^	0.148	0.132	0.132	0.076

^1^Kruskal Wallis ^2^Friedman **Bold** = Significant changes. T0 baseline, T1 after treatment, T2 3 months after treatment, T3 12 months after treatment. Bonferroni corrections have been conducted in relation to each analysis.

Finally, OB did not differ between the groups on any occasion. It increased significantly in the RTW and SL groups, Table 4.

**Table 4:** Occupational balance (OB) measured with the Occupational Balance Questionnaire (OBQ) from T0 to T3 in participants with depression and anxiety disorder

	OB. T0 (OBQ)	OB. T1 (OBQ)	OB. T2 (OBQ)	OB. T3 (OBQ)	*P* value within
	Median Min–Max	Median Min–Max	Median Min–Max	Median Min–Max	samples^2^
Sick leave (*n* 24)	20 4–44	26 7–55	29 1–47	32 7–56	**<0.01**
Return-to-work (*n* 13)	25 6–35	31 10–47	38 9–51	44 17–54	**<0.01**
Working (*n* 17)	29 14–46	34 15–50	36 5–50	42 10–50	0.057
*P*-value between samples^1^	0.284	0.632	0.240	0.900

^1^Kruskal Wallis ^2^Friedman **Bold** = Significant changes. T01 baseline, T1 after treatment, T2 3 months after treatment, T3 12 months after treatment. Bonferroni corrections have been conducted in relation to each analysis.

## 4 Discussion

The present study aimed at describing and comparing QOL, SOC, and OB over time in a sample of people with depression and anxiety disorders, who differed in relation to their paid work situation. The participants were analyzed separately depending on whether they had been on sick leave continuously, returned to work or been in work continuously for one year after the occupational therapy treatment they had participated in.

No statistically significant changes between the groups were identified at any time. The pattern, however, indicates that the participants in the SL group consistently rated lower than the participants in the RTW and Work groups. Moreover, all groups improved over time, but the SL group did so to a lesser degree. The SL participants solely improved their OB whereas the RTW group improved their QoL and their OB, and the Work group improved their QoL and SOC. This result shows some similarities to that in our previous study [20] in the present sample; where the focus was on their perception of their performance and satisfaction with the performance on valued activities in everyday life. The SL group in that study improved in fewer activity areas, socialisation and work, compared to the RTW (improved in the areas active recreation, socialisation and work) and Work (improved in the areas active recreation, household, personal care and socialisation) groups. However, considering that the previous study [20] focused on their everyday activities it is somewhat surprising that the sole improvement in the SL group in the present study was an increased level of OB, which also has a focus on everyday activities. No information is available for their rationale for their ratings and further studies with a qualitative design would thus be relevant. Moreover, a study focusing on the relationship between performance and satisfaction with the performance of activities and the perception of the right mix of activities in everyday life (occupational balance) would also be relevant.

Both the RTW and the Work groups increased their ratings of QOL [35] over time. This is similar to previous study results of increased QOL after an intervention [13, 23, 24, 42], although these studies did not include a subgroup division between participants differing in relation to involvement in paid work. The present study thus contributes with showing the possibility that the improvement varies between different subgroups of participants.

SOC [38] increased over time among the Work group participants but not in the other groups. Improvements after treatment have been shown in similar populations in previous studies [26, 27], but no subgroup division of the participants in relation to their paid work situation had been conducted. It should, however, be recognized that the time span of the previous studies [26, 27] is mostly similar to the time span between T0 to T1 in the present study. Furthermore, although the RTW group did not improve significantly over time and did not statistically significantly differ from the other groups in SOC, they showed the largest variation in SOC over time as their median was 20 points higher at T3 (median 66) than at T0 (median 46). The result in the present study thus has both some similarities to and differences from results in previous studies with other populations [28–30], which found a statistically significant higher level of SOC in those returning to work.

The SL and RTW groups rated a significant increase in OB [39] over time but to a different extent with a higher median in the latter group. The higher median in the Work group over time was also close to being statistically significant. The ratings of OB in the RTW and Work groups at T3 are also similar to previous results in studies in general populations [41, 43], which might indicate that these participants experience more of an adequate level of OB over time.

In summary, the results indicate the relevance of dividing participants in accordance with their paid work status for gaining more information about variations within the group of clients with depression and anxiety. These variations can perhaps also relate to their rehabilitation needs. Further studies with larger samples are, however, warranted. Moreover, no knowledge exists concerning the participants’ rationale for their ratings. Future studies combining self-ratings with qualitative interviews into a mixed method approach, would thus be valuable for further investigations as there may be a number of factors that can have impacted on the participants’ ratings. A recent literature review focusing on sick leave in people with depression described the causes of longer sick leaves. These were related to the disorder, the work place as well as to the individual him/herself [44]. Some gender differences during RTW, such as women reporting home-related demands more than men do [10] have also been described. No knowledge exists about such issues among our participants. However, it should also be noted that having a choice of whether and when they should return to work was not available to all of them, e.g., due to not receiving benefits for sick leave [45].

### 4.1 Limitations and strengths

The present study has both methodological limitations and strengths, which need to be considered when interpreting the results. The small number of participants in the groups is considered to be a severe limitation. Further studies with larger numbers of participants are thus warranted for investigating whether the results of the present study can be generalized. Furthermore, no conclusion can be drawn about the potential effect of occupational therapy since the study did not involve any participants who did not receive occupational therapy. Further studies also need to be conducted that include participants who are not receiving occupational therapy but other forms of treatment in order to investigate the possible benefit of occupational therapy for this population.

It should also be noted that the participants in the SL group were older than those in the other groups and also rated higher levels of depression and anxiety with the Hospital Anxiety and Depression scale [33, 34] but potential implications of this were not investigated. Finally, it would have been valuable if more information had been collected about events that had occurred during this period, which could have influenced the participants’ perspective, e.g., about return to work, potential work accommodations [46] as well as about their rationale for various ratings.

However, the study had a longitudinal design and used valid and reliable instruments over time, which can be considered to be a strength for the internal validity. The internal consistency could also be considered adequate [47] in the present sample. The fact that all project assistants had been trained and validated prior to the data collection is also considered as strengthening the internal validity. Due to the number of analyses, the decision was taken to conduct Bonferroni corrections [48] in order to decrease the risk for a Type I error, which could thus be considered a strength. However, it should be recognized that this procedure may instead increase the risk for a Type II error.

## 5 Conclusion

Participants with depression and anxiety have previously been found to increase their quality of life, sense of coherence and occupational balance after rehabilitation. The results of the present study, with a very small sample, indicate that these aspects may vary between people who differ in terms of their paid work situation. The results can be considered an indication of the potential relevance of also dividing and studying participants with depression and anxiety separately in relation to their paid work situation.

## Ethical approval

The project was approved by the Regional Ethical Review Board in Linköping, Sweden (Dnrs. 2012/232-31 and 2015/12-32).

## Informed consent

All participants signed an informed consent form.

## Conflict of interest

The authors have nothing to declare.
